# A conversation on the impacts and mitigation of air pollution

**DOI:** 10.1038/s41467-021-25491-w

**Published:** 2021-10-04

## Abstract

Air pollution is an environmental and health concern affecting millions globally every day. Professor Denise Mauzerall, an expert in air pollution and climate change at Princeton University, shares with *Nature Communications* their thoughts on the impacts of air pollution and the policies needed to tackle emissions.

1. What aspect of air pollution concerns you the most?

I am most concerned that air pollutant and greenhouse gas (GHG) emission mitigation be addressed simultaneously in order to protect both human health and climate at the same time. Such strategies provide substantial technical and cost co-benefits.

**Figure:**
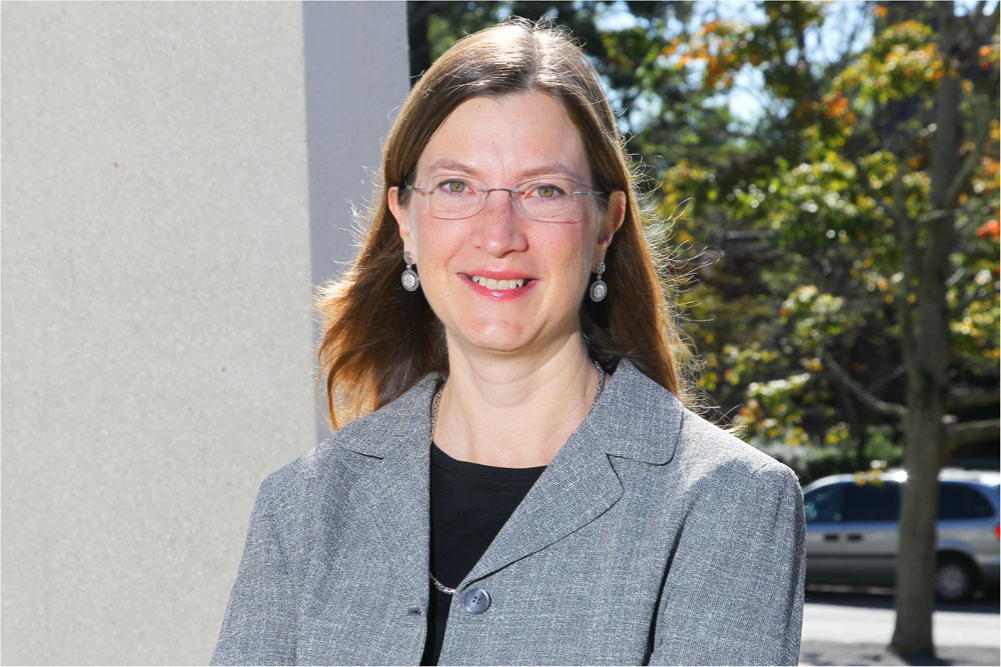
Denise Mauzerall.

Over the long-term, climate change will worsen air pollution, even in areas where it has been improving. Climate change is resulting in hotter drier conditions in many parts of the world which is leading to increased frequency and intensity of wildfires. In the western U.S., climate change is causing record breaking temperatures, a widespread state of extreme drought, and an intense start to the fire season with 2021 fires outpacing those of 2020, itself a record breaking fire year. Wildfires release large quantities of smoke which is carried long distances by wind. Smoke from western fires reach across the country, affecting air quality in both urban and rural locations far from the fire location. In the eastern U.S. we have recently been smelling smoke from the western fires, experiencing very high levels of air pollution, and having particularly colourful sunsets brought on by the particulates in the smoke.

Smoke includes fine particulates (e.g. PM2.5, particulate matter with radius of 2.5 μm or smaller), hydrocarbons and nitrogen oxides. These compounds, and the photochemical smog they form, has adverse impacts on public health including increasing rates of premature deaths. Extreme heat and air pollution interact both chemically and within the human body making vulnerable people, and even otherwise healthy people, especially susceptible to their combined effects.

In 2020, annual average PM2.5 concentrations in the U.S. were heavily affected by smoke as well as by environmental rollbacks during the Trump administration. These factors led to PM2.5 concentrations exceeding World Health Organization standards in 38% of cities in the U.S. in 2020 compared with ~20% in 2018 and 2019.

Without dramatically reducing the emissions of greenhouse gases (GHG), forecasts indicate that fire frequency and intensity will continue to increase leading to widespread increases in air pollution and its associated impacts on human health. Thus to improve air quality, in addition to directly controlling the emissions of air pollutants, it is critical to reduce emissions of GHG in order to reduce future climate change and associated emissions of air pollutants from fires.

2. Given that air pollutant and GHG emissions may often originate from the same source, should we be simultaneously addressing reducing air pollutant and GHG emissions, and how could this be achieved?

Climate change and air quality should no longer be considered separate issues. It is imperative to mitigate greenhouse gas (GHG) and air pollutant emissions simultaneously. Research in my group has highlighted many opportunities to do so. Since most air pollutant and GHG emissions come from combustion, energy sources that minimize or eliminate combustion can reduce emissions of both. Electrification of vehicles and the use of hydrogen as a vehicle fuel could greatly reduce the emissions of both air pollutant and carbon emissions, but only if the electricity and hydrogen that is used in these substitutions is largely produced from increasingly non-fossil energy like renewably generated electricity or nuclear power. However, if the electricity and hydrogen comes from conventional coal and gas sources, tail-pipe emissions of air pollutants will be reduced, but the upstream emissions of air pollutants and GHG could increase and will remain too large for us to avoid dangerous levels of climate warming. In addition, the use of electric heat pumps for heating and cooling buildings, the reuse of waste heat in industry, and improved energy efficiency in all sectors will benefit air quality, health and climate. Going forward, both sets of emissions should be considered together as simultaneous mitigation has long-term economic benefits as well as providing immediate benefits for air quality, health, climate and ecosystems.

3. What are your thoughts on current policy enforcement, and how well or not this is being achieved?

Enforcement of air pollution and other environmental regulations slackened under the Trump administration. Former President Trump weakened or eliminated key regulatory initiatives reducing air pollutant and GHG emissions started by the Obama Administration and withdrew the U.S. from the Paris climate agreement. In contrast, immediately after taking office President Biden identified a large number of clean air and carbon issues as top environmental priorities for his administration. In his first days in office, Biden re-entered the Paris agreement and signed an executive order directing agencies to examine dozens of Trump-era environmental rollbacks of clean air and carbon emission regulations, and Clean Air Act rules limiting the interpretation of scientific findings and restricting the inclusion of benefits not directly targeted by a regulation in evaluations of a regulations costs and benefits.

In addition, President Biden has set a target of ~50% reduction of GHG emissions from 2005 levels by 2030 and set a net zero economy-wide GHG emissions target of 2050. Proposed initiatives will reduce air pollutants and improve public health as well as decreasing emissions of GHG. Initiatives include increasing electrification of the transportation sector which will reduce tailpipe emissions of air pollutants from vehicles, decarbonizing the power sector which will decrease air pollutant emissions from power plants and hence further decarbonize downstream applications that use electricity, and broadly increasing energy efficiency which will decrease air pollutant and GHG emissions per unit service provided. Of course, these initiatives may be weakened by political compromises before implementation and will face severe challenges in Congress and the courts.

The Biden administration is also now reinstating a variety of environmental rules that benefit both air quality and climate. For example, methane is a potent short-lived greenhouse gas that also contributes to the formation of ozone, a criteria air pollutant with adverse health impacts. They are also reinstating rules that limit the emissions of methane from oil and gas extraction and is proposing funding to reduce emissions from abandoned oil and gas wells, something that my group identified as a significant emitter of methane for decades after the wells are abandoned.

4. How effective is voluntary action vs government mandated policy in reducing air pollution?

Government has a critical role to play in reducing air pollution and carbon emissions. Voluntary action, although important, is inadequate. Although over 50 companies across the economy have made climate pledges to have net-zero carbon emissions by 2040, most companies do not have the right economic incentives to make commitments at this time. Their commitments are not enforceable, and do not directly address emissions of health damaging air pollutants. Individuals do not have the ability to require low emissions from the products they buy or the activities in which they engage, although consumer choice and social norms can play a limited role in driving popularity of preferred products. Government policy and effective enforcement are critical. For example, corporate average fuel efficiency (CAFE) and air pollutant emission standards are government regulations that have been well enforced and have dramatically decreased emissions of GHG and air pollutants from vehicles and industry.

Under the Clean Air Act (CAA), the Environmental Protection Agency (EPA) sets limits on the allowed concentrations of certain pollutants in ambient air. EPA also regulates emissions of air pollutants from stationary sources (e.g. power plants and industry), mobile sources (e.g. vehicles) as well as the emission of air toxics, and enforces allowed limits. EPA’s controls via the CAA have been highly cost effective at dramatically improving air quality in the U.S. and protecting human health and welfare while supporting innovation and economic growth.

5. Socioeconomic factors such as income, education and wealth have been shown to play a key role in public health air pollution impacts. What needs to be done to ensure that policies developed are equitable and just?

In the United States marginalized groups, particularly communities of colour, have faced larger impacts from environmental threats. Socioeconomic inequalities have led to unequal investment in neighbourhoods, which has resulted in some areas becoming disproportionately burdened by emissions from nearby polluting power plants, industry and high traffic roads, resulting in higher levels of air pollution in those communities. Increased adoption of renewable energy would reduce emissions from the power sector and electrification of the transport and residential sectors would further reduce emissions in marginalized communities, increase the well-being of their residents and increase property values. Improvements in urban planning so that all communities have carbon-neutral transit options including walking and public transportation would help assure that sources of dirty air don’t concentrate in such neighbourhoods. Federal, state and local level government action can all help achieve these goals.

Inequalities are greatest in developing countries where many of the poor still rely on solid fuels for heating and cooking. Such residential use of solid fuels results in indoor exposure to high levels of health damaging fine particulate pollution as well as to elevated pollution levels in nearby neighbourhoods and regions downwind. Government support for improved stoves and cleaner fuels like electricity and natural gas, has already greatly reduced these emissions in northern China. According to our research, government support of electric heat pumps in the rural residential sector in China will provide the largest long-term opportunity of any heating option to improve air quality and decrease GHG emissions as the electric grid decarbonizes. Similar action in other less developed countries would also be beneficial.

6. Technological advances to mitigate air pollution such as retrofitting coal-fired plants are touted as potentially cost-effective solutions. What are the most promising recent advances to mitigate against pollutants?

The most promising recent advances to mitigate both air pollutants and GHG are economy wide electrification with non-fossil energy. This includes decarbonizing electricity generation with renewable energy and transforming the transport, residential and industrial sectors to be powered primarily by electric power. Conventional end-of-pipe emission controls on coal fired power plants reduce their emissions of health damaging air pollutants but do nothing to eliminate GHG emissions. End-of-pipe emission controls are costly and are a reason that many coal fired power plants in the U.S. have chosen to close. Closure of these coal plants has resulted in a large part of the decrease in U.S. carbon emissions over the past fifteen years. Adding pollution controls to coal power plants results in them becoming more expensive to operate than newly sited renewably generated electricity. As the cost of renewably generated electricity and battery storage continues to decrease, renewable energy will increasingly displace coal generation leading to large decreases in air pollutant emissions. Coal-fired electricity generation in the United States has decreased over 60% since 2008. It supplied only 23% of electricity demand in 2021 and is projected to supply only about 15% in 2026. If coal continues to be used beyond the near future, the implementation of carbon capture technologies around the world will be necessary to reduce their enormous adverse impacts on climate.

7. Do you hold out more hope for technological solutions, or political action, as a means to reduce air pollution?

Both technological and political solutions are needed. Technology forcing, for example via a carbon tax or fee for air pollutant emissions, can encourage increased efficiency and the uptake of pollution free technologies. Government funding is necessary to support basic and applied research to identify and develop technologies that reduce air pollutant and GHG emissions. Industry incentives to research and deploy air pollution and carbon free energy technology at scale would also be beneficial. Appropriate legislation and regulatory action to support government research, industry deployment, and private sector uptake of cleaner technology is critical. Public participation in the processes that result in legislation and regulation is valuable to spur initial uptake of the new technologies, continue to drive down costs, and shift social norms to prefer the newer cleaner technologies. Societal concern to reduce both air pollution and climate change can help motivate government action to support innovation and deployment of new technology.

8. Finally, how would you like collaboration between physical, health and policy scientists working on air pollution to improve?

When I arrived at Princeton as a young professor over twenty years ago, most research fell within disciplines. However, collaboration across disciplines seemed vital to solve pressing environmental problems. I set out to bridge disciplines in order to better inform environmental policy making. I was trained as an atmospheric scientist and spent several years in Washington DC working for an environmental consulting firm and the U.S. EPA. In Washington I came to realize that cooperation between government, industry and academia was vital for the development and deployment of sound environmental policy. At Princeton my research has involved collaborations with other atmospheric scientists, economists, political scientists, epidemiologists, agronomists, and technology experts around the world. Our collaborations have led to findings indicating that many opportunities exist to simultaneously decrease air pollutant and GHG emissions in ways which improve public health and are economically beneficial. Examples include increased electrification of the transport and residential sectors with decarbonized electricity, increased nitrogen use efficiency in agriculture so as much fertilizer as possible enters crops and as little as possible is lost to air and water, and decreases in international and bilateral financing of coal fired power plants that would decrease emissions of air pollutants and GHG.

Today, additional research collaborations between academia, government, and the private sector that identify mitigation strategies that have benefits for both air quality and climate is needed. Involvement of stakeholders in research would be beneficial. A focus on “sustainability” rather than independent efforts on air, land, water and climate pollutants is critical. Ambitious climate policies to limit global average increases in temperature to less than 2C above preindustrial levels, as the world has agreed to do in the Paris climate agreement, will have enormous benefits for air quality. The critical linkages between climate policy, air quality and public health and the potential for achieving co-benefits for these three factors via decarbonizing the global energy system (including technical, financial, and policy analysis) and improving global agriculture needs to be better quantified and brought more into public discussion. Increasing inter-disciplinary collaborations on climate change impacts and GHG mitigation has the potential to motivate a critical rapid transformation of current energy and agricultural technology and policy which will result in large immediate co-benefits for air quality and public health and a better future for our planet.

*This interview was conducted by Melissa Plail*.

